# Development of a Modular Tensegrity Robot Arm Capable of Continuous Bending

**DOI:** 10.3389/frobt.2021.774253

**Published:** 2021-11-01

**Authors:** Shuhei Ikemoto, Kenta Tsukamoto, Yuhei Yoshimitsu

**Affiliations:** Department of Human Intelligence Systems, Graduate School of Life Science and Systems Engineering, Kyushu Institute of Technology, Kitakyushu, Japan

**Keywords:** musculoskeletal robot, tensegrity, continuum robot, soft robotics, modular design

## Abstract

In this study, we present a tensegrity robot arm that can reproduce the features of complex musculoskeletal structures, and can bend like a continuum manipulator. In particular, we propose a design method for an arm-type tensegrity robot that has a long shape in one direction, and can be deformed like a continuum manipulator. This method is based on the idea of utilizing simple and flexible strict tensegrity modules, and connecting them recursively so that they remain strict tensegrity even after being connected. The tensegrity obtained by this method strongly resists compressive forces in the longitudinal direction, but is flexible in the bending direction. Therefore, the changes in stiffness owing to internal forces, such as in musculoskeletal robots, appear more in the bending direction. First, this study describes this design method, then describes a developed pneumatically driven tensegrity robot arm with 20 actuators. Next, the range of motion and stiffness under various driving patterns are presented as evaluations of the robot performance.

## 1 Introduction

As a feature of the biological body that is expected to be applied to robots, musculoskeletal system has garnered considerable attention in the literature. The musculoskeletal system is generally overdriven because muscles can only generate tension. This means that some of the tension in a muscle is consumed as an internal force to balance the tension in other muscles. The function of these antagonistic pairs is to alter the joints stiffness, and coordinate postural changes in response to external forces. Several musculoskeletal robots have been developed to provide robots with functionalities, i.e., bio-inspired embodied intelligence (Pfeifer et al., 2007), generated by this redundancy. In particular, the challenge of realizing a complex three-dimensional musculoskeletal system like that of living organisms in robots has been continuously pursued in this field (Kozuki et al., 2016; Kurumaya et al., 2016; Tadesse et al., 2016; Hitzmann et al., 2018). However, it is tough to realize the complex musculoskeletal robot that is practical and applicable for various tasks because several features are difficult to realize by engineering, such as multi-degree-of-freedom joints, soft tissues, and their lubrication. Therefore, to understand and apply bio-inspired embodied intelligence, which arises from the redundancy of the complex musculoskeletal system, it is necessary to take an approach that is not limited to mimicking the actual musculoskeletal system. As one of the approaches for this purpose, we leverage tensegrity in this study.

Tensegrity originally refers to a stable three-dimensional structure comprising struts that bear compressive forces and cables that bear only tensile forces, but where the struts are not connected (Burkhardt, 2008). It has begun to attract attention in architecture (Gomez-Jauregui, 2004), and is now attracting widespread attention in biology (Ingber, 2003a,b), engineering (Sultan, 2009), and information science (Caluwaerts et al., 2013). Using this broad spread across multiple disciplines, the term “tensegrity” has been utilized in recent years, even if it does not meet all of the aforementioned definitions. Specifically, even if the struts are not bar-shaped and bear bending stresses, or even if there are connections between rigid bodies, they are often called tensegrity if they are stable three-dimensional structures under cables tension. In this study, hereafter, the former will be referred to as the strict tensegrity, and the latter as the broad tensegrity.

Research and development of robots utilizing tensegrity have been actively pursued in robotics, and both strict and broad tensegrities have been leveraged. As tensegrity robots that employ the strict tensegrity, deformable and rolling spherical robots have been studied for years (Rieffel et al., 2007; Shibata and Hirai, 2009; Kim et al., 2014, 2017; Vespignani et al., 2018). The strict tensegrity comprises an insignificant number of parts, which are lightweight and robust, and can be made flexible and actively deformable by introducing springiness and actuators to some of the cables. This is a valuable feature in robots for locomotion, where interaction with the environment is inevitable. Therefore, there have been active studies on its control (Caluwaerts et al., 2014; Kim et al., 2020), and the utilization of data-driven approaches has gained significant attention in recent years (Kim et al., 2015; Zhang et al., 2017; Zhu et al., 2018; Surovik et al., 2019). However, there are also studies utilizing broad tensegrity for this type of tensegrity robot. The utilization of bent bars instead of struts to improve the shape and deformation during movement is one of the approaches utilizing broad tensegrity (Rhodes et al., 2019; Schorr et al., 2021a). In this case, the bent bar bears the bending stress, which is not entirely the strict tensegrity, but the bent bar can be regarded as a straight strut; hence, it approximately follows the definition of strict tensegrity. This means that the bent bar must be thicker and heavier for strength, but other advantages of the strict tensegrity are maintained, such as flexibility to the entire structure. In addition, as a tensegrity robot for another kind of locomotion, a tensegrity robot that stores and releases elastic energy for jumping has been developed (Schorr et al., 2021b). This robot cannot even be interpreted as the strict tensegrity, and does not benefit from its inherent advantages. Meanwhile, it is easy to design a mechanism that solely allows specific movements by limiting deformation locations. The ease of design to obtain a target mechanism is the main reason to utilize the broad tensegrity instead of strict tensegrity for tensegrity robots.

The fact that the broad tensegrity is helpful to pursue a specific function is not limited to locomotion. For instance, the broad tensegrity is utilized to realize multi-degree-of-freedom joints such as the shoulder joint (Wang and Post, 2021) and spinal structures Sabelhaus et al. (2020); Zappetti et al. (2020). A similar trend is also determined in manipulators utilizing tensegrity structures. Most of the manipulators utilizing tensegrity can be classified into two types: those that utilize multiple struts connected like rigid body parts (Lessard et al., 2016; Jung et al., 2018; Ramadoss et al., 2020), and those that utilize linkage mechanisms by connecting struts with a single degree of freedom joints (Fadeyev et al., 2019; Fasquelle et al., 2020). In these studies, it is clear that they are not utilizing the strict tensegrity, but the broad tensegrity. In addition, there are tensegrity manipulators in which a few struts are replaced with compression springs (Wei et al., 2020). This mechanism is widely utilized in continuum manipulators (Rao et al., 2021), and it cannot also be regarded as the strict tensegrity because it includes either a member that bears the bending moment, or a joint that reduces the degree of freedom.

The above indicates that the broad tensegrity has been utilized for manipulators, while the strict tensegrity has not been utilized. This is because the broad tensegrity easily achieves desired non-spherical shapes and mechanisms, while the strict tensegrity is difficult to do the same. However, to increase redundancy and flexibility, it is desirable to utilize the strict tensegrity, which reduces the number of parts, allows several actuators to be placed without interference, and provides flexibility to the entire structure. The further away from the strict tensegrity, the more the inherent features of the strict tensegrity are lost. Therefore, as the system’s flexibility and redundancy increase, similar implementation challenges are thought to eventually appear as in tendon-driven systems utilizing rigid joints (e.g., musculoskeletal systems).

This study proposes a design method for an arm-type tensegrity robot with a long shape in one direction, and can be deformed like a continuum manipulator. This method is based on the idea of utilizing flexible and straightforward strict tensegrity modules, and connecting them recursively so that they remain strict tensegrity even after being connected. The tensegrity obtained by this method strongly resists compressive forces in the longitudinal direction, but is flexible in the bending direction. Therefore, the changes in stiffness owing to internal forces, such as in musculoskeletal robots, appear more in the bending direction. This study describes this design method, then describes a developed pneumatically driven tensegrity robot arm with 20 actuators accordingly. As evaluations of the availability of the robot, the range of motion and stiffness under various driving patterns are presented.

## 2 Proposed Design Rule

**FIGURE 1:**
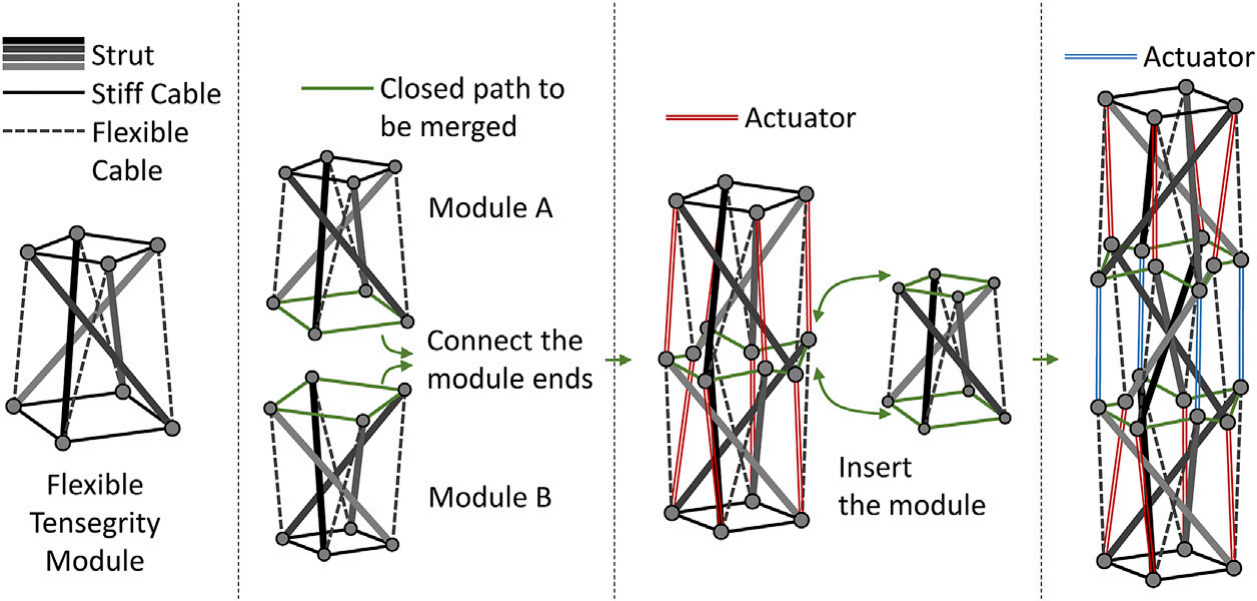
Proposed design rule of a tensegrity robot arm. The tensegrity module constituting the tensegrity robot arm has two types of cables: a stiff cable and a flexible cable. Because the flexible cables allow deformation of the module, two modules can be connected by the closed path composed of stiff cables. To make the connected structure a tensegrity, each end on the closed path needs an additional connection with an end belonging to the different module, not being on the closed path, and does not have an opposite neighboring end. The proposed design rule utilize actuators for these additional cables. The addition of a third or subsequent module is considered to be the insertion of the module into the closed path connecting two modules. To keep this a tensegrity, cables that connect ends on the two new closed paths, but don’t belong to the inserted module are needed. These additional cables mean actuators in the proposed method similar to the two module connections.

Figure 1 illustrates the overview of the design rule proposed for tensegrity robot arms in this study. This design rule is based on the idea of connecting simple modules to obtain an overall complex tensegrity. Here, a simple module refers to a strict tensegrity that can be designed intuitively by the designer. The tensegrity to be utilized as a simple module does not require any structural complexity such as the number of struts and cables, but it requires the following two characteristics:

1) It must have stiff cables that can be observed as unchanging in length, and constitute at least one closed path.
2) It must have flexible cables whose length can be altered according to applied tension, allowing passive deformation of the entire structure while still having feature 1.

The first characteristic is for specifying the paths that connect simple modules. Because the structure of the connected modules is an arm-like tensegrity long in one direction, this characteristic also suppresses the change in length in the longitudinal direction. The second characteristic is for allowing the deformations required when simple modules are connected. The longitudinal length change is suppressed, while the curved posture is allowed in the arm-like tensegrity. In particular, Figure 1 illustrates an example of such a simple module, comprising four struts, and having two closed paths.

Simply connecting two modules to share a closed path of stiff cables does not make the structure tensegrity. To make the connected structure have tensegrity, each end on the closed path needs an additional connection with an end belonging to the different module, not on the closed path, and does not have the opposite end neighboring. As illustrated in Figure 1, the proposed design rule utilizes actuators for those additional cables. Aforementioned, because the arm-like tensegrity is solely allowed to deform in the bending direction, because of the closed path of stiff cables, the active deformation of the tensegrity by the actuators appears as bending. To distribute the actuators uniformly, the connection of the third and subsequent modules is according to a different rule, which inserts a module into the already connected closed path. In particular, to keep the structure after inserting a tensegrity, actuated cables that connect ends on the two new closed paths but not belonging to the inserted module are introduced. Consequently, the arm-like tensegrity appears to have a similar number of layers as the connected modules, and each layer/module will have a similar number of actuators.

To validate the basic idea of the proposed design rule, we fabricated a mock-up in which the actuators were implemented with flexible cables. This model is an arm-like tensegrity comprising five modules connected by the proposed design rule, which belongs in the strict tensegrity. In this model, the stiff cables are the PE lines for fishing, and the flexible cables are implemented by attaching a spring in the middle of the PE lines. By observing the passive deformation when an external force is applied, we can verify that the tensegrity fabricated by the proposed design rule can be continuously bent, and that it will be able to bend when actuators apply internal force actively.

**FIGURE 2:**
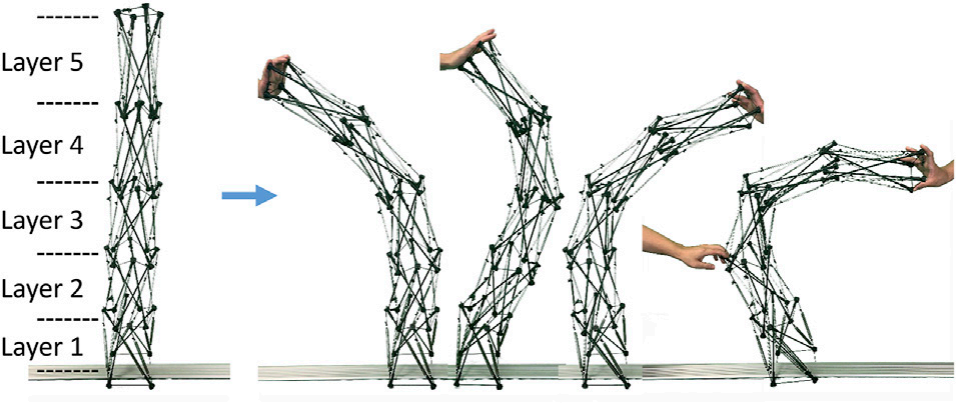
The tensegrity robot arm mock-up fabricated by the proposed design rule and the verification results. The mock-up was fabricated utilizing a simple tensegrity comprising four struts as a module, and connecting five of them based on the proposed design rule. Each of the five modules can be viewed as a layer of the arm-like tensegrity. By applying an external force, a variety of continuous bending postures can be observed. As expected, the closed path comprising stiff cables suppresses changes in the longitudinal length, and external forces contribute solely to changes in the bending direction.

Figure 2 illustrates the overview of the mock-up and the result of the verification. From Figure 2, first, it can be confirmed that the proposed design rule provides an arm-like tensegrity. Because five modules are connected, we can observe five layers corresponding to them. Because the module is a tensegrity comprising four struts, as illustrated in Figure 1, four actuators per layer should be implemented, but flexible cables should replace them in this mock-up. Next, it can be observed that the arm-like tensegrity takes various continuous bending postures when an external force is applied. This indicates that the closed path comprising stiff cables play a role in suppressing the longitudinal length change as expected. Therefore, we can expect that the active posture changes by actuators will also result in a similar continuous bending behavior.

## 3 Developed Tensegrity Robot Arm

**FIGURE 3:**
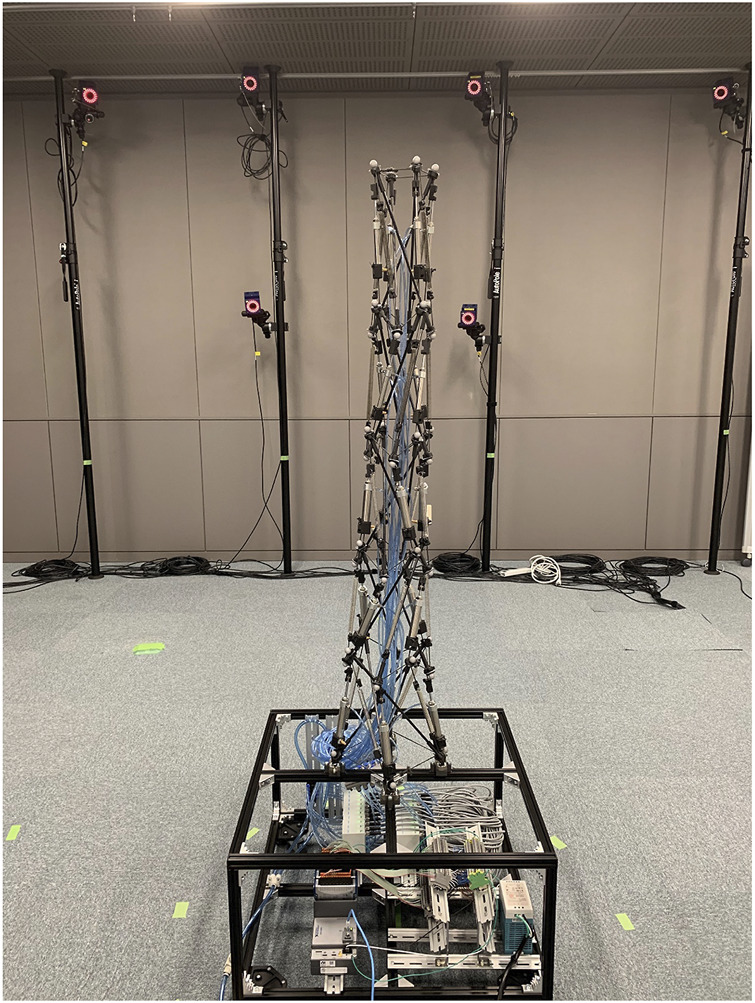
The overview of the developed tensegrity robot arm capable of continuous bending. In this robot, five tensegrity modules are connected by the proposed design rule, and 20 pneumatic cylinders are employed as actuators. Note that two types of modules with different twisting directions are utilized, and they are connected alternately. The instruments for the control system are implemented in the box frame that the robot is mounted.

By utilizing pneumatic cylinders as actuators for the five-modules arm-like tensegrity (see Figure 2) constituted by following the proposed design rule (see Figure 1), we developed the tensegrity robot arm capable of continuous bending. Figure 3 illustrates the overview of the developed robot. The robot arm incorporates 20 double-acting pneumatic cylinders arranged according to the proposed design rule. As illustrated in the picture, the robot arm is mounted on a black aluminum frame box, which contains the pressure control valves, the built-in controller, and other devices for the control. The size of the base box is 0.6 (m) × 0.6 (m) × 0.3 (m), the height of the whole arm is 1.2 (m) without the box, and the mass of the whole arm including the air tubes is 2.9 (Kg). In the following subsections, we provide detailed information on the mechanical structure and the control system.

### 3.1 Mechanical Structure

The entire structure of the developed tensegrity robot arm is the strict tensegrity. Therefore, the structure can be constructed by repeatedly utilizing few parts such as struts, cables, spring-loaded cables, and actuated cables.

**FIGURE 4:**
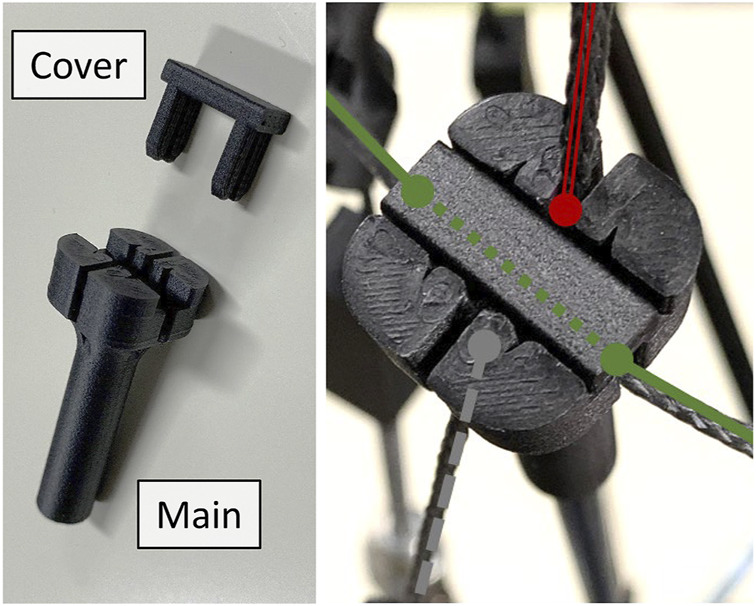
The developed cable fixing part attachable at the ends of struts. The fixing part comprises the body part and the cover. The body part has slits in four directions for attaching the cable, and the clamps or knots on the cable can be caught in the gaps to fix the cable firmly. Because there are closed paths with stiff cables in the proposed design rule, as an alternative, it allows the stiff cables to pass through the body part *via* slits, and the cover can firmly fix it. These parts are fabricated by the FDM 3D printer (Mark Two, Markforged).

All struts are made of CFRP pipes of 5 (mm) in outer diameter and 300 (mm) in length, and cable fixing parts are attached to both ends of the pipes. Figure 4 illustrates the cable fixing part that we developed. As illustrated in this figure, the body part has slits in four directions for attaching the cable, and the clamps or knots of the cable can be caught in the gaps to fix the cable firmly. Because stiff cables that shape closed paths are utilized in the proposed design rule, as an alternative way, the stiff cable can pass through the body part and be firmly fixed by the cover. This cable fixing part makes connecting the modules according to the proposed design rules easier. To fabricate these parts, we utilized an FDM 3D printer (Mark Two, Markforged). This 3D printer utilizes a nylon material mixed with short carbon fibers, and place long carbon fibers for reinforcement in the layering plane, thus achieving the accuracy and strength required for cable fixing parts. The spring-loaded and actuated cables are realized by connecting springs and pneumatic cylinders between the stiff cables, respectively. Those connecting parts for these types of cables were also fabricated utilizing a similar 3D printer. Because of the cable fixing parts at the ends of struts and these connecting parts for spring-loaded and actuated cables, the entire structure of the tensegrity robot arm is established without utilizing any screws, except for the nuts at the rod ends of the pneumatic cylinders.

Two types of springs with equal unloaded lengths of 80 (mm) and different spring constants are utilized for the modules. Specifically, the lower three modules utilize springs with a spring constant of 0.55 (N/mm) (AUS10-80, Misumi), and the upper two modules utilize springs with a spring constant of 0.22 (N/mm) (AWY10-80, Misumi). Similarly, two types of pneumatic cylinders with an equal stroke length of 45 (mm) and different diameters are utilized for the actuators. Specifically, the lower three layers utilize cylinders with a diameter of 16 [mm] (MSPCN16-45, Misumi), and the upper two layers utilize cylinders with a diameter of 10 (mm) (MSPCN10-45, Misumi).

In the developed tensegrity robot arm, two types of four-struts tensegrity modules with different twisting directions are utilized, and they are connected alternately. It is possible to utilize only modules with a similar direction of torsion, but in this case, the torsional stiffness of the entire arm in that direction will be lower than that of the opposite direction. This causes the twisting of the entire arm to slightly appear when the tip of the arm in an upright position is pushed downward. In the developed tensegrity robot arm, modules twisted in different directions are connected alternately to reduce this effect, and the twist is not observed even when the tip is pushed down.

### 3.2 Control System

**FIGURE 5:**
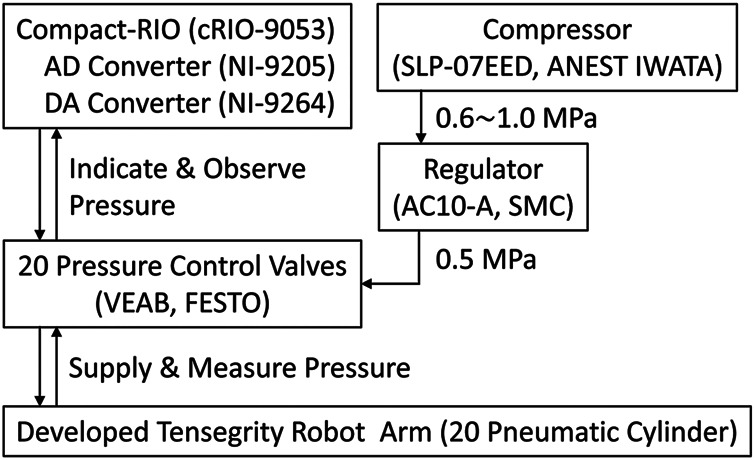
The schematic of the control system. Pressure control valves supply compressed air to the pneumatic cylinders and measure the actual pressures in their chambers. The pressure control valves are connected to the embedded control device having AD/DA converter modules. Also, stable compressed air of 0.5 MPa is supplied to the pressure control valves. The base box of the developed tensegrity robot arm encloses the pressure control valves and the embedded control device.

Figure 5 illustrates the schematic of the control system. Because solely contraction force is required for all actuators, proportional pressure control valves (VEAB, FESTO) are connected solely to the contraction ports, where cylinders contract when pressurized, and only silencers are attached to the other ports. Accordingly, the control system utilizes 20 pressure control valves. The control valve receives and outputs the desired and actual pressures as analog voltages, respectively. These analog signals are read/written using the analog-to-digital converter (NI-9205, National Instruments) and the digital-to-analog converter (NI-9264, National Instruments) so that the embedded control device (cRIO-9053, National Instruments) mounted in the base box can observe/control the robot arm.

**FIGURE 6:**
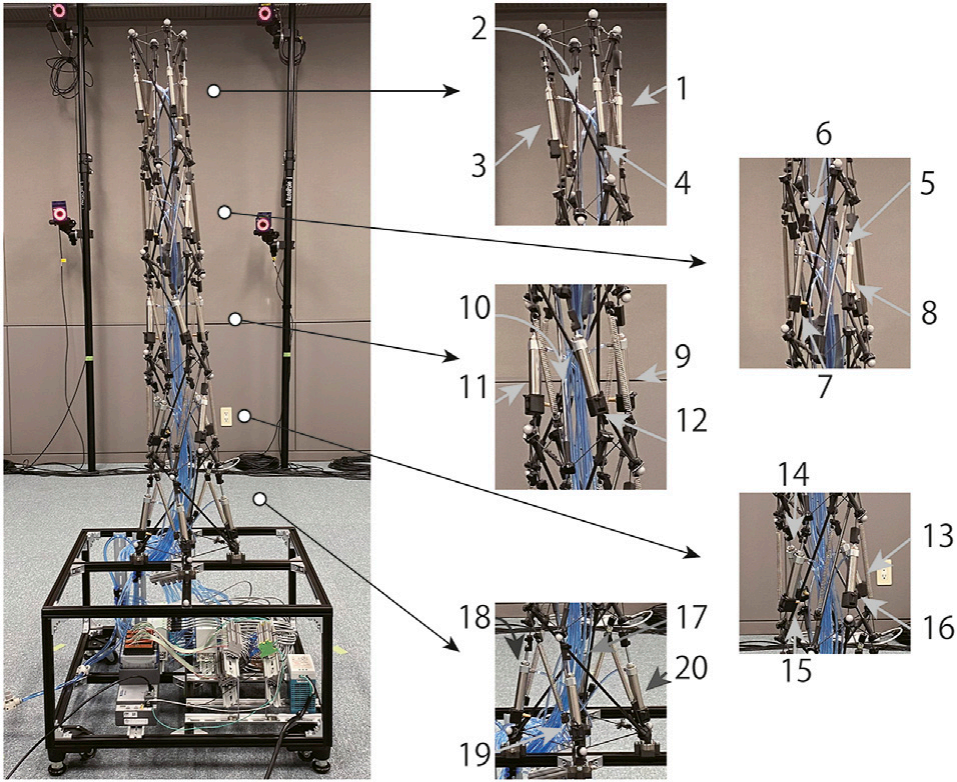
The numbering rule for 20 pneumatic cylinders in the developed tensegrity robot arm. The pneumatic cylinders are numbered counterclockwise in each layer, starting from the cylinder at the extreme back right. All the 20 pneumatic cylinders are numbered by following this procedure for all five layers from the tip to the base.

The 20 pneumatic cylinders are numbered from the top to the bottom layer, and the four pneumatic cylinders in each layer are numbered in a counterclockwise direction, starting with the cylinder located in the extreme back right. Figure 6 illustrates this numbering rule for 20 cylinders. By controlling the contraction force of these cylinders, the arm moves to a posture where the axial forces of all the members are balanced.

The challenge of finding a form in which the axial forces of all members are balanced is one of the significant challenges in the field of tensegrity, which is called the form-finding challenge (Sultan et al., 2002; Zhang and Ohsaki, 2006). Because the form-finding challenge is closely related to the control of tensegrity robots (Kim et al., 2015, 2020), developing the kinematic controller based on the form-finding challenge is valuable also for the developed tensegrity robot arm, which is categorized as the strict tensegrity. However, in this study, we focus on the development and evaluation of the tensegrity robot arm itself, to enable our control system solely implement the function of manually providing the desired values of the internal pressure of the 20 pneumatic cylinders; hence, the robot arm is controlled in a feedforward manner.

## 4 Experiment

To verify that the developed tensegrity robot arm can be utilized as a flexible and redundant robot platform with qualitative features of musculoskeletal robots, we conducted experiments to evaluate the range of motion and stiffness. In the experiments, postures of the developed tensegrity robot arm were measured utilizing the optical motion capture system (MAC3D System, Motion Analysis).

**FIGURE 7:**
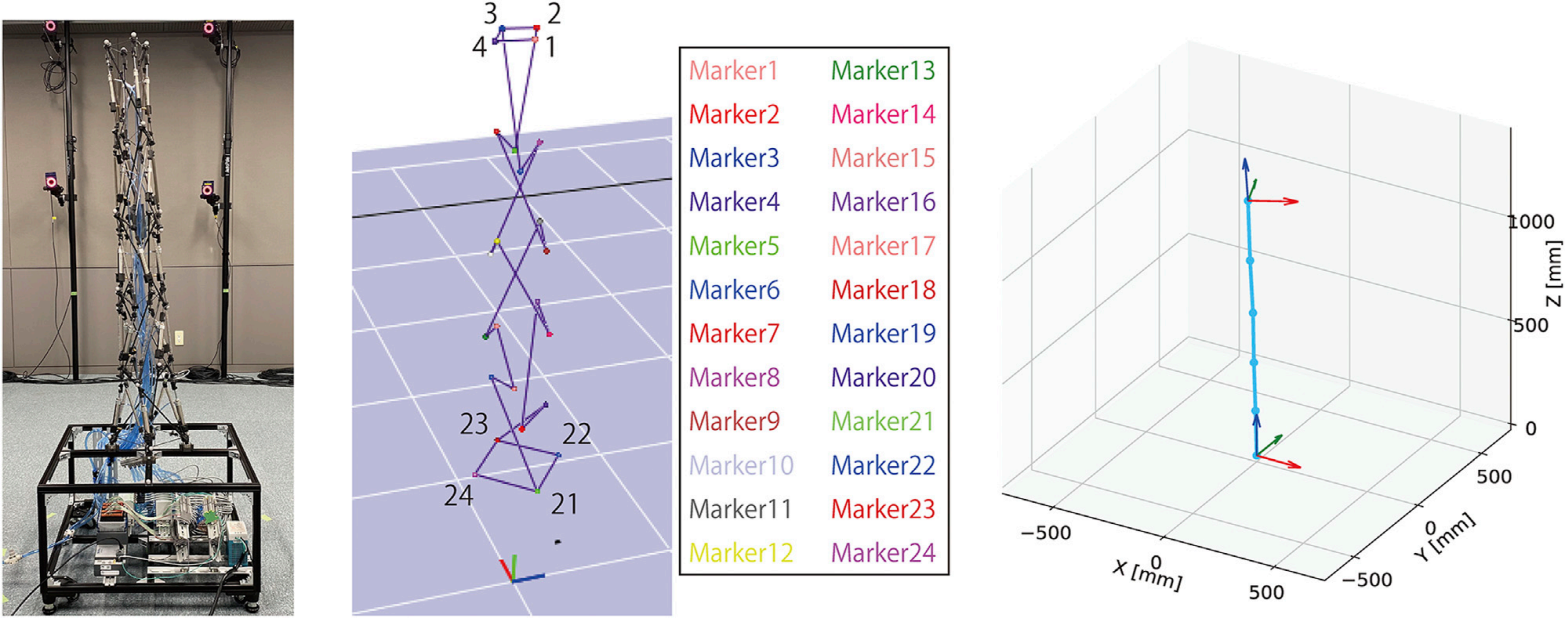
The overview of the measuring system utilizing the optical motion capture system. A total of 24 markers, four for each of the six closed paths of stiff cables, are attached to the developed tensegrity robot. The centers of the six closed paths are calculated from the marker positions, and the robot posture is expressed, i.e., a serial link mechanism with five links connected by multi-degree-of-freedom joints. The base coordinate system is defined as a right-handed system, with the origin at the bottom closed path’s center. The tip coordinate system, which eases understanding the posture, is defined as a right-handed system. The Z and X axes are defined as the normal of the least-squares plane of marker 1 to marker 4, and the projection of marker 2 onto the least-squares plane, respectively.

Figure 7 illustrates the overview of the measuring system. As illustrated in the figure, 24 markers of the optical motion capture system are attached to the developed tensegrity robot arm. The robot has six closed paths of rigid cables, and the center positions of the closed paths are calculated from the four markers on each closed path. These center positions represent the posture of the entire robot arm, which is a serial link mechanism with five links connected by multi-degree-of-freedom joints. The base coordinate system of the robot is defined as a right-handed system, with the origin at the closed path’s center at the bottom. However, the hand-tip coordinate system is defined with the closed path’s center at the top as the origin. The X-Y plane is first defined as the least-squares plane of markers 1 to marker 4, to determine the normal, i.e., the Z-axis. The projection of marker 2 onto the least-squares plane next defines the orientation of the X-axis, and the remaining Y-axis is defined so that the entire system is right-handed. In this study, this tip coordinate system is utilized to quickly understand the posture and twist of the whole arm from the measurement results.

**FIGURE 8:**
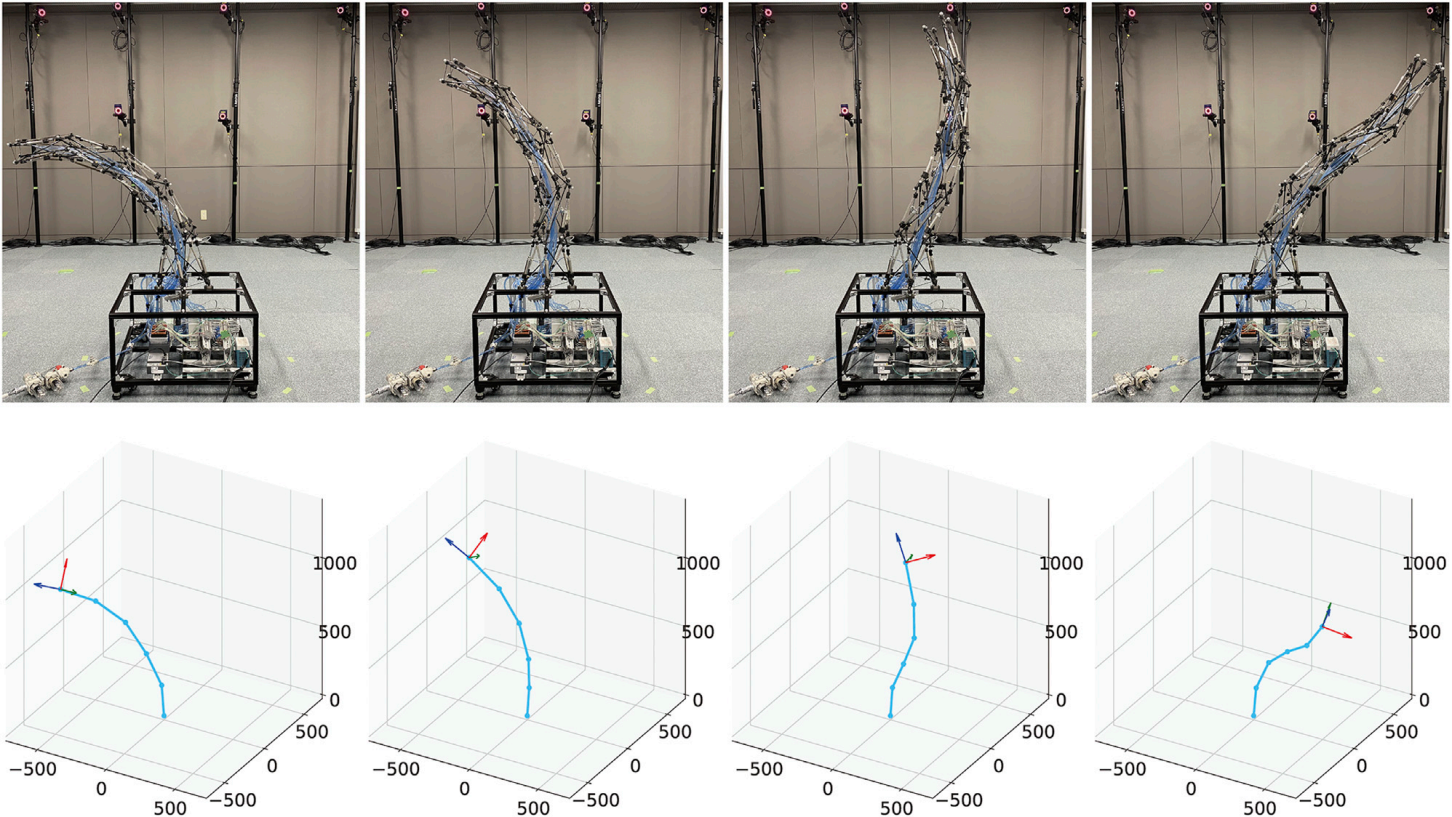
The examples of postures and their corresponding measurements. The developed tensegrity robot arm can not only bend in one direction, but also in different directions twice. The postures are successfully measured by the measuring system utilized in this study.

Figure 8 illustrates examples of postures and their corresponding measurements. As illustrated in this figure, it can be observed that the developed tensegrity robot arm can take a variety of continuous bending postures, and the optical motion capture system can successfully measure them. In the following subsections, we explain the evaluation of the movable area, and the variable stiffness feature of the developed tensegrity robot.

### 4.1 Evaluation of Movable Area

To evaluate the range of motion, we generated 1,024 different 20-dimensional vectors that mean sets of desired pressures for pneumatic cylinders. The pressure vectors were generated with the following deterministic procedure like a grid sampling:

1) Define 0 (MPa), 0.25 (MPa), and 0.5 (MPa) as discrete desired pressure values, meaning no drive, weak drive, and strong drive.
2) Prepare four types of four-dimensional vectors representing the desired pressure values of the four pneumatic cylinders for each layer. These vectors mean to command 0.5 (MPa), 0.25 (MPa), 0 (MPa), and 0.25 (MPa), starting clockwise from one of the four pneumatic cylinders in that layer.
3) Generate 1,024 types of 20-dimensional vectors from combinations of five layers.

**FIGURE 9:**
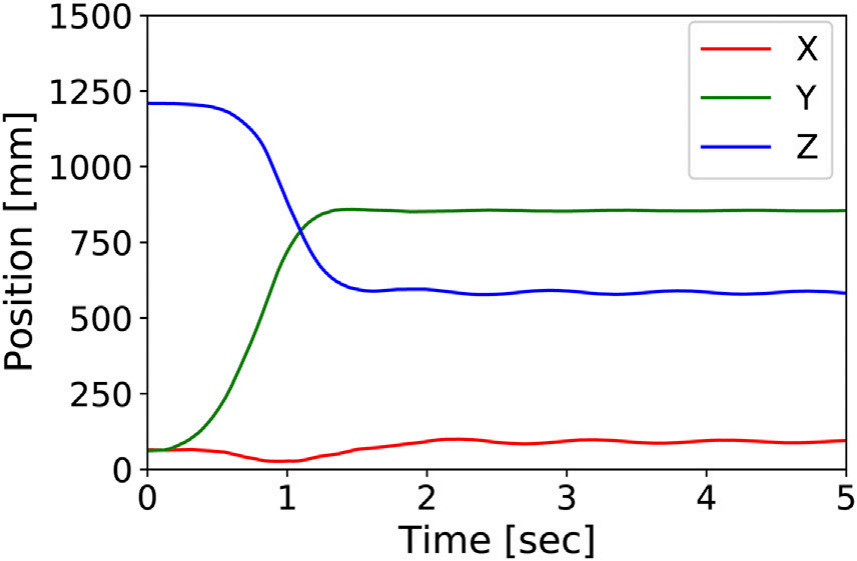
The time series of the hand-tip position during a motion to the maximum bending posture. It shows that the steady-state is reached in about 5 s. Supplementary Video S1 shows this motion.

For all the 1,024 desired pressure values, the posture of the developed tensegrity robot arm was measured after waiting for 5 s to reach the steady-state after input. Figure 9 shows the time series of the hand-tip position during a motion to the maximum bending posture. Supplementary Video S1 shows this motion.

**FIGURE 10:**
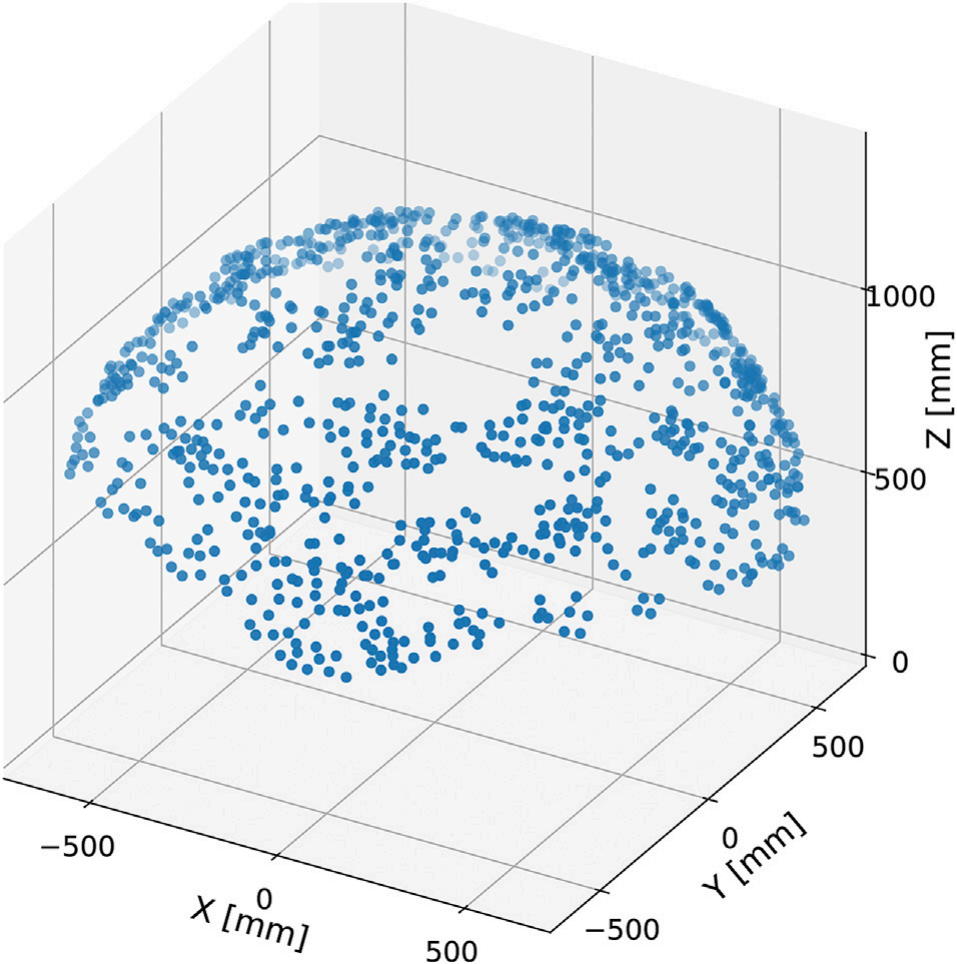
The scatter plot of the tip positions of the developed tensegrity robot arm. The definition of the coordinate system corresponds to that illustrated in Figure 7. The tip position is widely distributed in a hemispherical manner. If viewed in the horizontal plane, the distribution is isotropic. The maximum/minimum tip positions are −857 (mm)/803 (mm) in the *x* direction, −777 (mm)/854 (mm) in the *y* direction, and 581 (mm)/1,206 (mm) in the *z* direction, respectively.

Figure 10 illustrates the scatter plot of the tip position, i.e., the center position of the top closed path of stiff cables, of the developed tensegrity robot arm in the base coordinate system. As illustrated in this figure, the range of motion is hemispherical and isotropic in the horizontal plane. Specifically, the tip of the developed tensegrity robot arm ranged -857 (mm) ∼ 803 (mm) in the *x* direction, -777 (mm) ∼ 854 (mm) in the *y* direction, and 581 (mm) ∼ 1,206 (mm) in the *z* direction.

**FIGURE 11:**
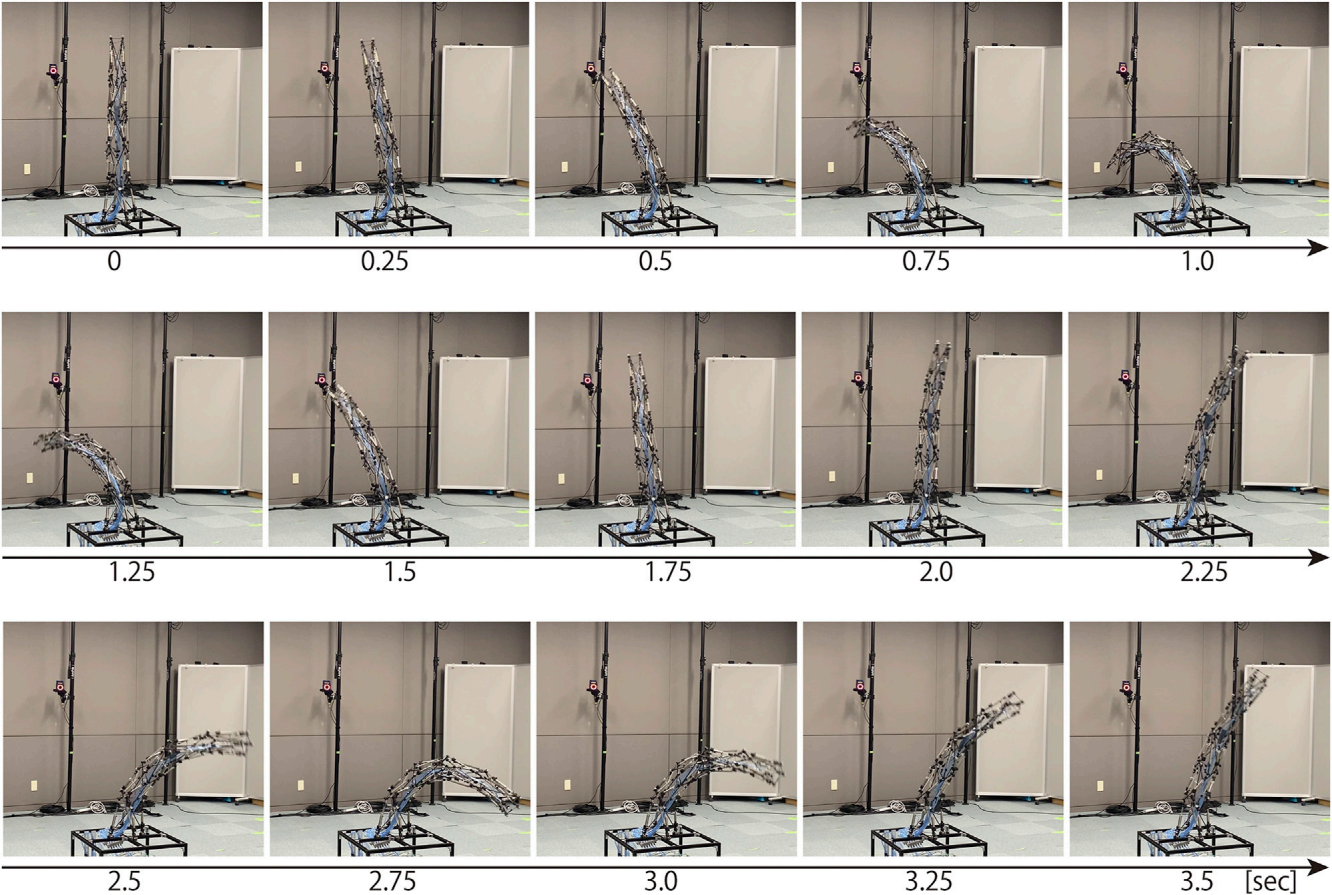
The sequential snapshots of the dynamic swing motion of the developed tensegrity robot arm. This swing motion was generated by grouping all the 20 pneumatic cylinders into ten cylinders each on similar and opposite sides, and giving each group a pulse wave with a maximum value of 0.5 (mm), a minimum value of 0.1 (mm), a duty ratio of 0.5, and a period of 3 (sec) in reverse phase. At 0 (sec), the robot arm was stationary, and these sequential snapshots indicate the beginning of the swing motion. Supplementary Video S2 shows this motion.

If the robot arm has a similar flexibility as the musculoskeletal system, the range of motion in the dynamic motions should be wider than that in the static motions. Next, we illustrate the dynamic motion of the robot arm, and compare the most bent postures with those in the static motions. Figure 11 illustrates sequential snapshots of the dynamic swing motion of the developed tensegrity robot arm. Supplementary Video S2 shows this motion. In this swing motion, 20 pneumatic cylinders were grouped into ten cylinders each on similar and opposite sides. Within each group, a similar series of desired pressure values were utilized, and the series was generated by a pulse wave with a maximum value of 0.5 (MPa), a minimum value of 0.1 (MPa), a duty ratio of 0.5, and a period of 3 (sec). The phase was determined so that another group utilizes the counter phase of a similar pulse wave. From this figure, it can be observed that the developed tensegrity robot arm can perform dynamic motions. Moreover, it is worth mentioning that the developed end part illustrated in Figure 4 was able to fix the cable firmly, and did not cause any challenge during this dynamic motion.

**FIGURE 12:**
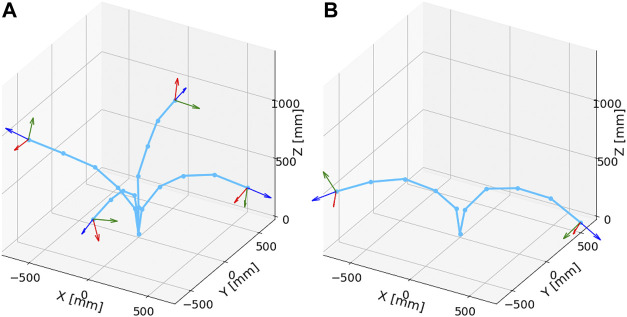
The comparison between most bent postures in the dynamic and static motions. **(A)**: Most bent postures in the static motions. From the postures of the tip coordinate system, it can be observed that there is no significant torsion in the robot arm. **(B)**: Most bent postures in the dynamic motions. The amount of bending is more significant than that in the static case. Furthermore, in contrast to the static motion, significant torsion in the robot arm can be observed.

Figure 12 illustrates the comparison between most bent postures in the dynamic and the static motions. These most bent postures of static and dynamic motions were determined from data in Figures 10, 11, respectively. This result indicates that the amount of bending in the dynamic motion is more significant than that in the static motion. In addition, from the postures of the tip coordinate system, it can be observed that the robot arm was significantly twisted in the dynamic motion in contrast to the static motion. Therefore, it is considered that the developed tensegrity robot arm has similar flexibility as the musculoskeletal system.

### 4.2 Evaluation of Variable Stiffness

**FIGURE 13:**
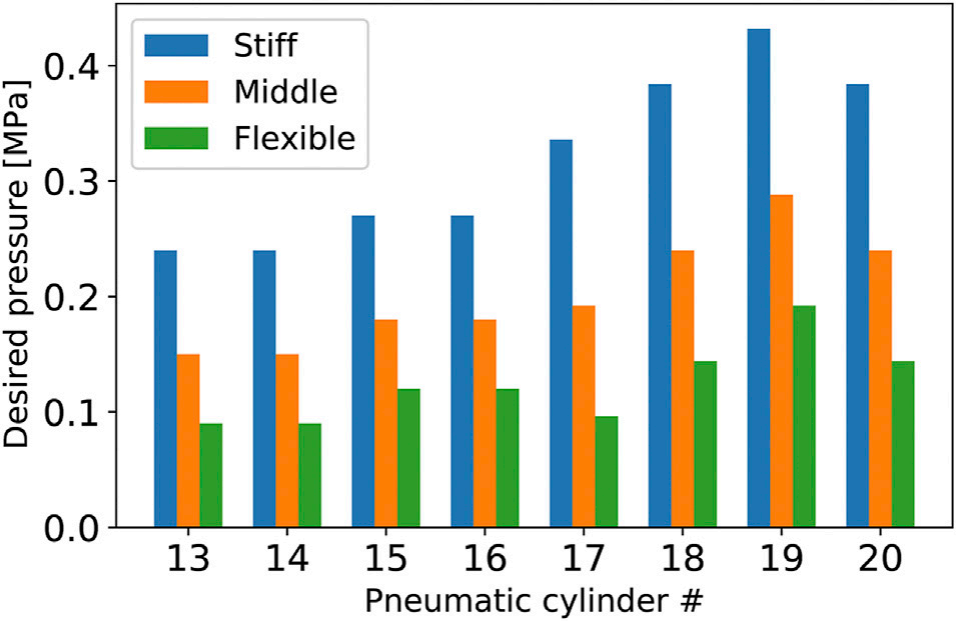
The three different sets of desired pressure values that result in almost the same posture of the developed tensegrity robot arm. The desired pressure values for pneumatic cylinders No. 1 to No. 12, which are not indicated in this graph, were set to (0.24, 0.48, 0.24, 0, 0.48, 0.48, 0, 0, 0.15, 0.3, 0.15, 0) (MPa), respectively, similarly for the three different sets. These values were configured manually.

The musculoskeletal system is overdriven, creating redundancy where several different inputs conduce a similar output. A typical function of this redundancy is a change in stiffness. In this section, we confirm that the developed tensegrity robot arm has such a variable stiffness. Consequently, we first prepared different desired pressure values that lead to an approximate similar posture of the robot arm. Figure 13 illustrates the three different sets of desired pressure values that result in an approximate similar posture. Because the desired pressure values for pneumatic cylinders No. 1 to No. 12 were set to similar values for the three sets, only the desired values for No. 13 to No. 20 are indicated in this graph. Specifically, the desired pressure values for pneumatic cylinders No. 1 to No. 12 were set to (0.24, 0.48, 0.24, 0, 0.48, 0.48, 0, 0, 0.15, 0.3, 0.15, 0) (MPa), respectively. This figure illustrates that three sets have different average pressures for pneumatic cylinders No. 13 to No. 20. Note that these values were configured manually.

**FIGURE 14:**
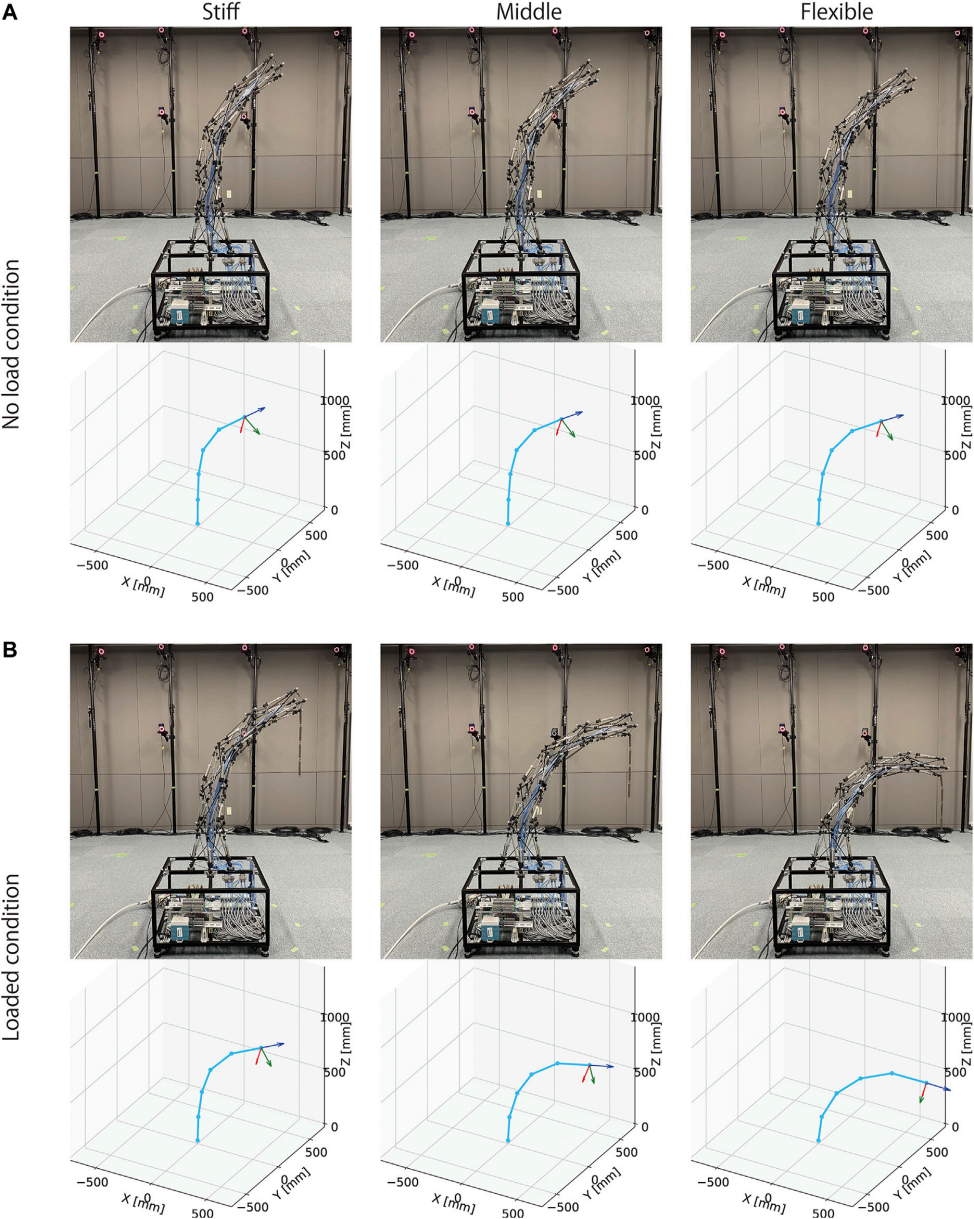
The postures of the tensegrity robot arm under no-load and loaded conditions. **(A)**: the postures by three different desired pressures illustrated in Figure 13 under the no-load condition. The three different desired pressures resulted in similar postures. The average tip position of the robot arm is (*x*, *y*, *z*) = (432.4, 78.7, 1037.3) and the standard deviation is (*STD*_*x*_, *STD*_*y*_, *STD*_*z*_) = (30.3, 37.6, 21.1). **(B)**: the postures by three different desired pressures illustrated in Figure 13 under the loaded condition where a 130 (g) mass was attached at the tip. The amount of bending was reduced by higher desired pressures, compared to that of lower desired pressures. The average tip position of the robot arm is (*x*, *y*, *z*) = (652.2, 178.2, 797.0) and the standard deviation is (*STD*_*x*_, *STD*_*y*_, *STD*_*z*_) = (81.0128.1147.2). These results indicate that the developed tensegrity robot arm has variable stiffness.

Figure 14 illustrates the postures and changes with loading conditions, when desired pressure sets illustrated in Figure 13 are provided. From this figure, preliminarily, it can be observed that the three different desired pressures resulted in an approximate similar posture in the no-load condition. Quantitatively, the average position of the center of the top closed path of the stiff cables is (*x*, *y*, *z*) = (432.4, 78.7, 1037.3), and the standard deviation is (*STD*_*x*_, *STD*_*y*_, *STD*_*z*_) = (30.3, 37.6, 21.1). However, under the loaded condition, where a 130 (g) mass was attached at the tip, these three different desired pressures exhibited distinctly different postures. From the pictures and graphs on the lower part of this figure, similar to a joint driven by an agonist-antagonist pair of muscles, it can be observed that stronger overall contractions of the pneumatic cylinders (i.e., higher desired pressures) increase the stiffness of the robot arm, and reduce the deformation caused by external forces.

**FIGURE 15:**
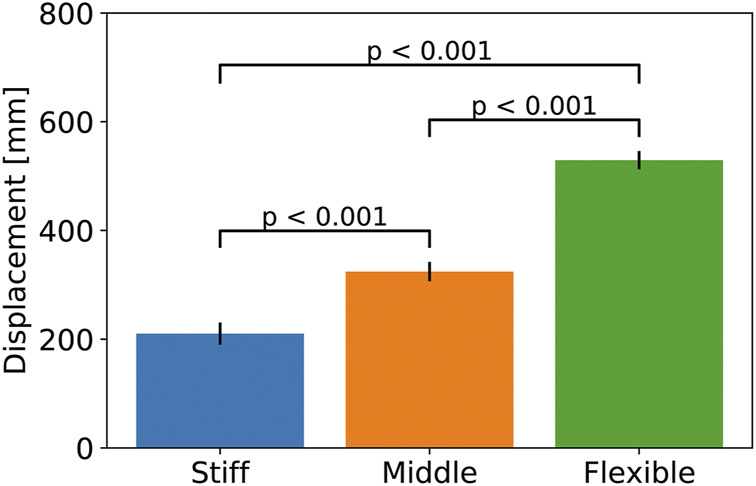
The comparison of displacements by the loading among three different desired pressures. Welch’s *t*-test was performed in the multiple comparison procedure among the three groups. The number of samples was 10 for each group. Each comparison shows *p* < 0.001, indicating a significant difference with a probability of significance of less than 1*%*, even after considering the Bonferroni correction.

Figure 15 shows the comparison of displacements by the loading among three different desired pressures. It indicates that displacements are different, and three different desired pressures lead to different stiffness as intended. To confirm statistically, we performed Welch’s *t*-test in the multiple comparison procedure among the three groups. The number of samples was 10 for each group. As a result, each comparison shows *p* < 0.001, indicating a significant difference with a probability of significance of less than 1*%*, even after considering the Bonferroni correction. These results mean that the robot arm exhibited a different type of bending in the loading condition, while postures were approximately similar in the no-load condition. Therefore, these results successfully confirm that the developed tensegrity robot arm has variable stiffness, such as musculoskeletal robots.

## 5 Discussion

In the design rules proposed in this study, the cables of each tensegrity module are defined as stiff cables constituting closed paths and springy cables. The closed path is necessary to prevent the arm from collapsing in the longitudinal direction, while the springy cable, which is passive, can be replaced by an actuator with series elasticity such as a pneumatic cylinder. Utilizing actuators that directly deform each module is promising for increasing the range of motion, because it can be difficult to significantly deform a module by driving the cables that cross between modules, depending on the posture. This improvement also leads to increased redundancy, which is highly compatible with the focus of this study.

A more obvious approach to increase the range of motion is to increase the number of modules. However, to increase the number of modules without altering the total length of the tensegrity robot arm, it is necessary to reduce the size of the module. The main challenge in reducing the size of the module is the displacement of the actuators. The contraction ratio, which means the amount of displacement relative to the maximum total length of the actuator, is generally less than 0.5 for linear actuators, and it decreases as the total length is reduced. Therefore, if the module is reduced in size, the amount of deformation of the module must be reduced. Winding cables by rotary actuators are one possible solution, but it still poses a mounting challenge because the clearance decreases as the module is reduced in size. In summary, to significantly increase the range of motion of the tensegrity robot arm, a comprehensive approach that covers the size and structure of the module, and the number and type of actuators is necessary.

Quantitative evaluation of the stiffness and payload of the developed tensegrity robot arm is crucial future work. As a preliminary evaluation, we measured the blocking force of the hand-tip by pulling in one direction from the maximum bending position to an approximately upright position. The result shows approximately 6 (N). However, because the tensegrity robot arm has high redundancy, the blocking force measurement essentially requires a suitable jig and multi-axis force/torque sensors. In addition, the strong dependence on posture also makes measuring the blocking force difficult. In parallel with improving the tensegrity robot arm, we will establish a measurement environment for quantitative evaluation of its capability.

This study has evaluated the developed tensegrity robot arm utilizing manually determined desired pressure values, but automatically generating those inputs is essential to utilize the tensegrity robot arm. We presume that the data utilized in this study’s analysis can be directly leveraged to model the forward kinematics utilizing machine learning. However, modeling inverse kinematics will be difficult because multiple sets of desired pressure values can realize a similar posture because of the high redundancy. In the future, we plan to utilize a method that simultaneously learns kinematics and inverse kinematics, and models redundancy as well (Masuda et al., 2019) to exploit the vital feature of the developed tensegrity robot arm.

## 6 Conclusion

In this study, we introduced a tensegrity robot arm, developed to reproduce complex musculoskeletal structures features. Preliminarily, a design rule for a tensegrity robot arm in which an entire structure is constituted by stacking simple tensegrity modules was proposed. Based on the proposed design rule, a tensegrity robot arm comprising five four-struts tensegrity modules was developed, and the technical details were explained. This robot arm is driven by 20 pneumatic cylinders, and can bend to various postures like a continuum manipulator. Utilizing an optical motion capture system, postures of this robot arm were measured in several experimental setups. By these experiments, the range of motion and the stiffness variation were evaluated. Accordingly, the fact that the developed tensegrity robot arm has similar features to musculoskeletal robots was successfully confirmed.

## Data Availability

The raw data supporting the conclusion of this article will be made available by the authors, without undue reservation.
